# Meta-Analysis of Whole Blood Transcriptome Datasets Characterizes the Immune Response of Respiratory Syncytial Virus Infection in Children

**DOI:** 10.3389/fcimb.2022.878430

**Published:** 2022-04-13

**Authors:** Qianyu Feng, Shujin Lin, Huifang Liu, Bin Yang, Lifen Han, Xiao Han, Lili Xu, Zhengde Xie

**Affiliations:** ^1^Beijing Key Laboratory of Pediatric Respiratory Infection Diseases, Key Laboratory of Major Diseases in Children, Ministry of Education, National Clinical Research Center for Respiratory Diseases, National Key Discipline of Pediatrics (Capital Medical University), Beijing Pediatric Research Institute, Beijing Children’s Hospital, Capital Medical University, National Center for Children’s Health, Beijing, China; ^2^Research Unit of Critical Infection in Children, Chinese Academy of Medical Sciences, Beijing, China; ^3^Mengchao Hepatobiliary Hospital of Fujian Medical University, Fujian, China; ^4^Vision Medicals Center for Infectious Diseases, Guangzhou, Guangdong, China; ^5^College of Biological Science and Engineering, Fuzhou University, Fujian, China

**Keywords:** respiratory syncytial virus, transcriptome, microarray, meta-analysis, immune response

## Abstract

Respiratory syncytial virus (RSV) is the most common and critical viral pathogen causing acute lower respiratory tract infection in infants and young children and has a huge disease burden worldwide. At present, there are many studies on RSV transcriptomics exploring the mechanism of disease, but different studies show different gene expression patterns and results due to different sample collection platforms and data analysis strategies. A meta-analysis was performed on eight whole blood transcriptome datasets containing 436 children with acute RSV infection and 241 healthy children. A total of 319 differentially expressed genes (DEGs) (P value <0.0001) were identified in a meta-analysis using a random effect model. Functional enrichment analysis showed that several pathways related to immunity were significantly altered, including the “chemokine signaling pathway”, “natural killer cell mediated cytotoxicity” and “cytokine–cytokine receptor interaction”. Immune cell type analysis showed that the proportion of neutrophils in most RSV-infected children was higher than that in healthy children. These immune characteristics may help to provide new insights into RSV infection in children.

## Introduction

Human respiratory syncytial virus (RSV), a negative sense, enveloped RNA virus, belongs to the *Pneumoviridae* family (Amarasinghe et al., 2019). Approximately all infants become infected with RSV by the age of 2 years, making it the leading viral pathogen of acute lower respiratory tract infections (ALRIs) in children (Glezen et al., 1986; Ge et al., 2018). It is estimated that more than 3 million hospitalizations in children within 5 years of age are associated with RSV ALRI per year globally, resulting in nearly 60,000 deaths (Shi et al., 2017). Although facing a huge disease burden, the current treatment remains limited to supportive treatment, and there is no safe and effective vaccine and antiviral drugs (Behzadi and Leyva-Grado, 2019). Therefore, it is necessary to further explore the pathogenic mechanism of RSV infection and provide a new understanding for the prevention and treatment of RSV.

The microarray is used as a common tool for transcriptomics analysis of differentially expressed genes (DEGs) and pathway exploration of disease-related mechanisms (Burel and Peters, 2018). However, the microarray results are often not repeatable, and even slight disturbances are unreliable due to different sample acquisitions, platforms, and inconsistent data analysis strategies, making different microarray studies on the same disease show inconsistent gene expression patterns and results (Cahan et al., 2007). The meta-analysis of microarray data has been proposed to greatly improve the gain of information obtained from microarray experiments and to summarize and analyse gene expression data from individual research through the statistical algorithm to determine the common “higher levels” transcript spectrum (Adams et al., 2008). In particular, the addition of large samples is expected to detect genetic differences in genes that may not be discovered in individual studies and minimize false-positive discovery rates (Adams et al., 2008).

In this study, a meta-analysis was performed of eight transcriptomic studies containing genome-wide expression profiles of whole blood samples from children with acute RSV respiratory tract infection and healthy children. In addition, transcriptome changes in children with RSV respiratory tract infection were characterized, which may provide new insights into pathogenesis.

## Methods

### Data Collection

The gene expression microarray data of respiratory RSV infection in children by the NCBI GEO database (https://www.ncbi.nlm.nih.gov/geo/) were searched. The keywords were “respiratory syncytial virus” and “children”. The inclusion criteria of the dataset were as follows: 1) studies containing human whole blood samples; 2) each dataset contained more than 3 samples; 3) analysis on the expression microarray platform; and 4) studies containing individual patient expression data. The details of the selected dataset are shown in **Table 1**.

**Table 1:** Details of the eight included datasets.

GEO accession	Samples Size		Sample Source	Platforms
	Control	RSV		
GSE105450	38	89^*^	whole blood	GPL10558 Illumina HumanHT-12 V4.0
GSE117827	6*^‡^*	4	whole blood	GPL23126 Affymetrix Human Clariom D Assay
GSE103119	38	16	whole blood	GPL10558 Illumina HumanHT-12 V4.0
GSE103842	12	62	whole blood	GPL10558 Illumina HumanHT-12 V4.0
GSE80179	52	27	whole blood	GPL10558 Illumina HumanHT-12 V4.0
GSE77087	23	81*^†^*	whole blood	GPL10558 Illumina HumanHT-12 V4.0
GSE38900	39	135	whole blood	GPL6884 Illumina HumanWG-6 v3.0 GPL10558 Illumina HumanHT-12 V4.0
GSE42026	33	22	whole blood	GPL6947 Illumina HumanHT-12 V3.0

^*^Children with RSV infection included 33 outpatients and 56 inpatients.

^†^Children with RSV infection included 20 outpatients and 61 inpatients.

^‡^Control subjects were children having ambulatory surgery for nonacute conditions.

### Meta-Analysis of Differential Gene Expression

The original expression spectra of the datasets were extracted, and the data were processed by R for statistical analysis. The results were meta-analysed using the random-effects model, and DEGs were determined according to “random P <0.0001”.

### Functional Enrichment Analysis of Differentially Expressed Genes

The DEGs identified in this meta-analysis were annotated by Gene Ontology (GO) and Kyoto Encyclopedia of Genes and Genomes (KEGG) using the annotation, visualization, and integrated discovery database (DAVID, https://david.ncifcrf.gov/), with a filter condition of P < 0.05.

### Evaluation of the Proportion of Immune Cells

To explore the potential relationship between immune cells in children with acute respiratory infection with RSV, the mRNA expression matrix was normalized, and the CIBERSORT algorithm (https://cibersort.stanford.edu/index.php) (Newman et al., 2015) with the original CIBERSORT gene signature file LM22 and 1000 permutations was used to estimate the content of 22 human immune cells in each dataset.

## Results

### General Overview of the Datasets

**Figure 1:**
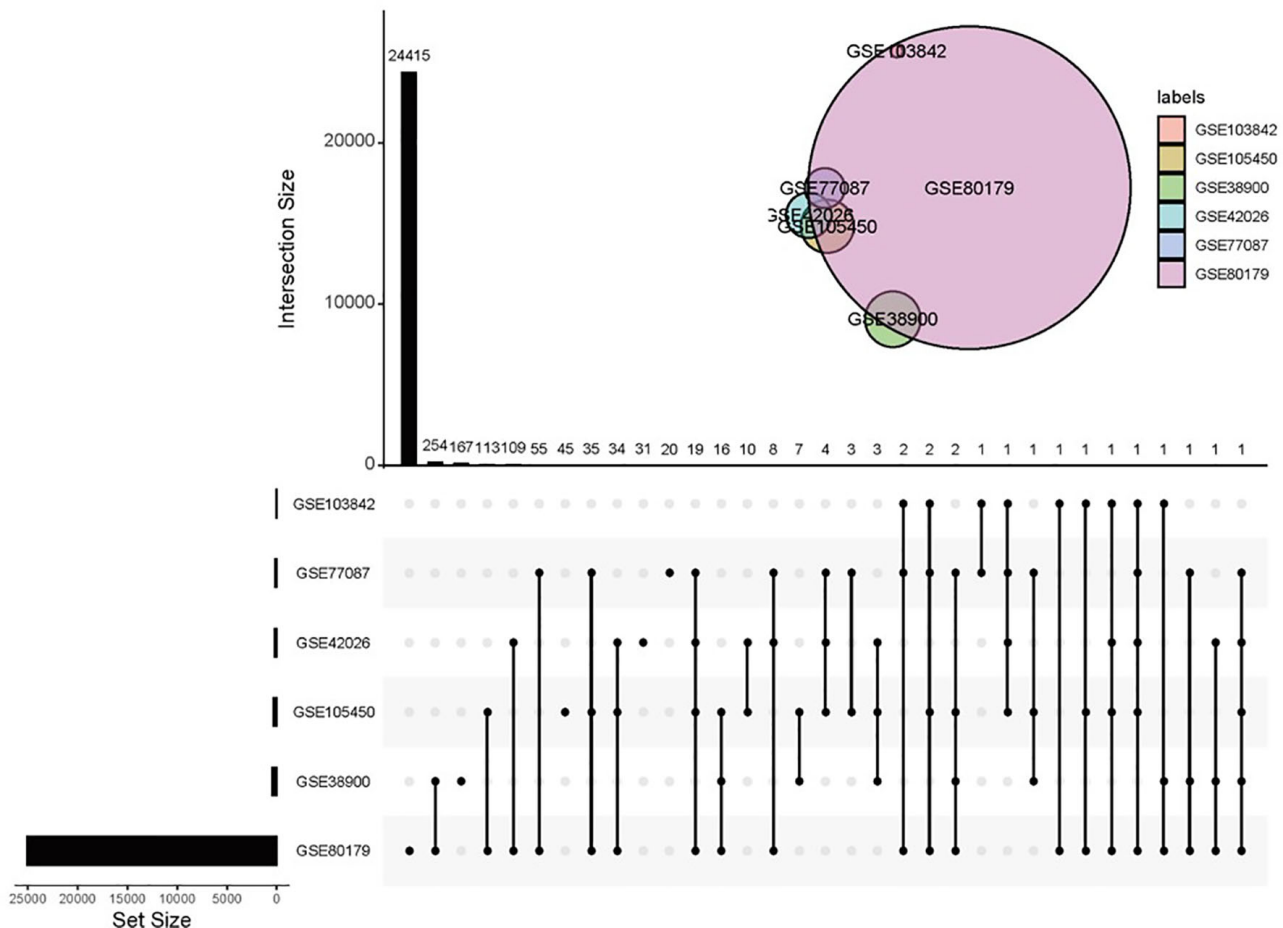
Overlapping DEGs in different studies. Note that there was no gene present in any of the six datasets in the UpSet diagram.

A total of 8 studies containing microarray expression data from 436 children with RSV infection and 241 healthy children were included in the meta-analysis to determine DEGs. Details of the selected datasets are shown in **Table 1**. All samples were obtained from patients’ whole blood. Six studies used the same microarray platform, GPL10558. GSE38900 had the largest sample size, including 135 children with RSV infection and 39 healthy children. In each study, the case groups consisted of children with acute RSV respiratory infection, and the control group consisted of healthy children except GSE117827, in which the control subjects were from children having ambulatory surgery for nonacute conditions. DEGs were screened from each study with q values less than 0.001 and fold changes greater than 2 indicating upregulation; fold changes less than 0.5 indicated downregulation as criteria. There were no DEGs screened out in GSE117827 and GSE103119. The UpSet diagram shows overlapping DEGs in six studies (**Figure 1**), with all DEGs identified by only meta-analysis rather than in individual analysis. The overlap between GSE38900 and GSE80179 was the largest, including 254 DEGs. Few genes (only two) were identified by the five studies. It was difficult to validate certain DEGs based on low experimental repeatability according to the traditional analysis method.

### Meta-Analysis of DEGs

To validate the DEGs according to different studies, we performed a meta-analysis on these transcriptome datasets. The eight studies were meta-analysed using a random effect model with 105280 genes detected. A total of 319 DEGs had a P value <0.0001 (**Table S1**), among which the expressions of 46 genes were upregulated and the expressions of 273 genes were downregulated. **Table 2** shows the top 20 most significantly upregulated and downregulated DEGs. *Sialic acid binding Ig-like lectin 1 (SIGLEC1)* had the largest upregulated TE value=1.42 (P value=1.29E-12), while *ribosomal protein S10 pseudogene 9 (RPS10P9)* had the largest downregulated TE value=-1.52 (P value=2.45E-13). The first three DEGs were *TPT1 pseudogene 4 (TPT1P4)*, *adhesion G protein-coupled receptor E4 (EMR4P)* and *ribosomal protein S7 pseudogene 11 (RPS7P11)*, with P value <5.00E-20 in each case.

**Table 2:** The top 20 most significant DEGs by meta-analysis.

Genes	P value
*TPT1P4*	2.96E-25
*EMR4P*	2.11E-23
*RPS7P11*	3.39E-20
*KRT72*	3.69E-20
*C1ORF151*	6.94E-20
*LOC729451*	3.48E-19
*RPS10P8*	1.05E-18
*RPS10P13*	3.84E-17
*NOV*	1.17E-16
*HS.578498*	2.13E-16
*KCTD14*	5.42E-15
*GPR44*	9.60E-14
*RPS27P23*	1.14E-13
*RPL9P32*	1.76E-13
*RPS10P9*	2.45E-13
*RPS10P4*	2.66E-13
*SIGLEC1*	1.29E-12
*PRSS33*	1.34E-12
*NFIA*	1.91E-12
*TPT1P4*	3.50E-12

### Functional Classification and Enrichment Analysis of DEGs

**Figure 2:**
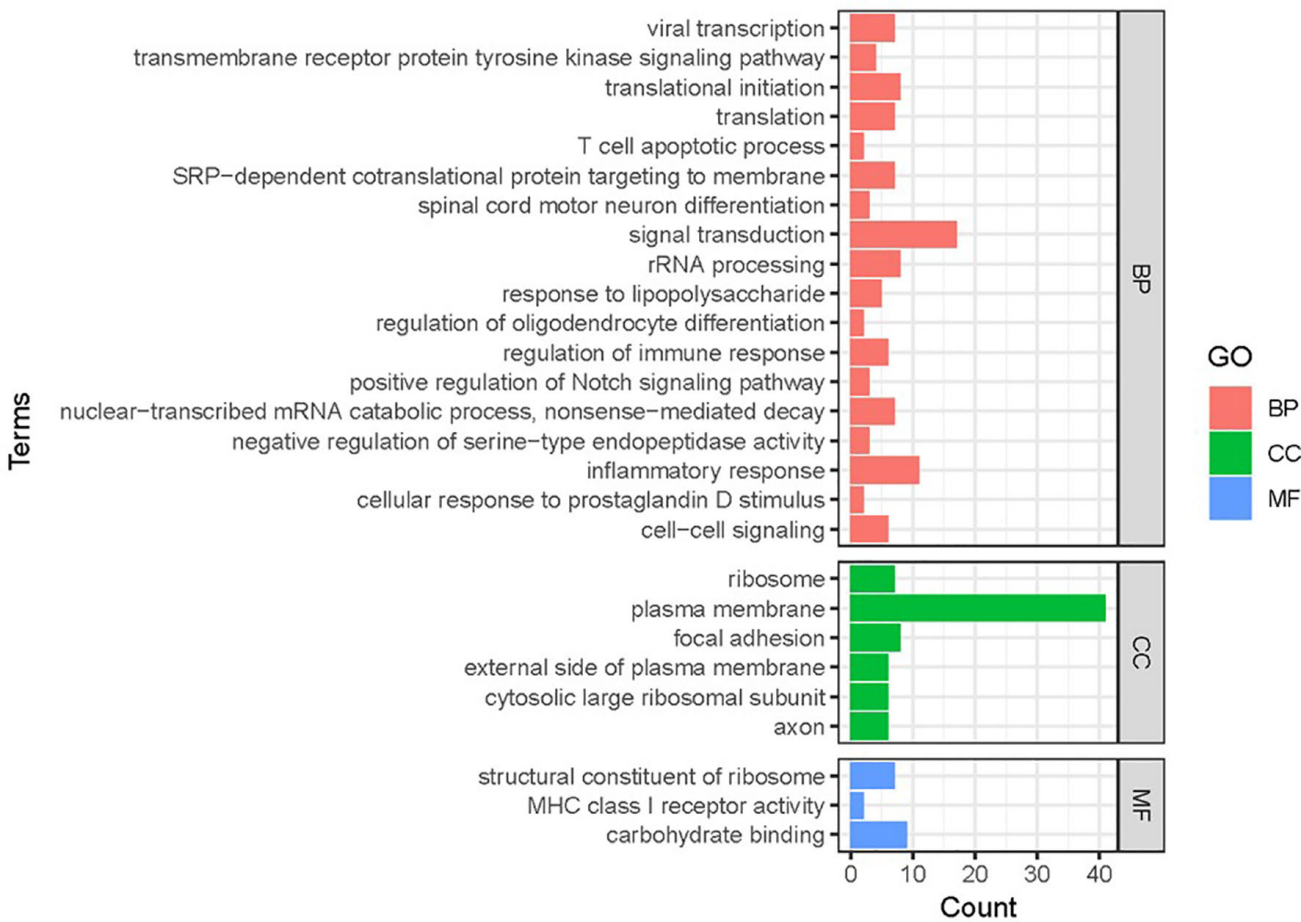
Gene functional classification of DEGs in RSV-infected children. BP, biological processes; CC, cellular compartment; MF, molecular function. Note that most DEGs belonged to “ribosomal processing” and “immune-related signaling”.

Gene functional classification analysis was performed to study the function of DEGs (**Figure 2**). Based on molecular functions, most DEGs mapped to ribosomal structural components and immune molecules. The DEGs were mainly involved in biological processes related to signal transduction, inflammatory response and translation.

**Figure 3:**
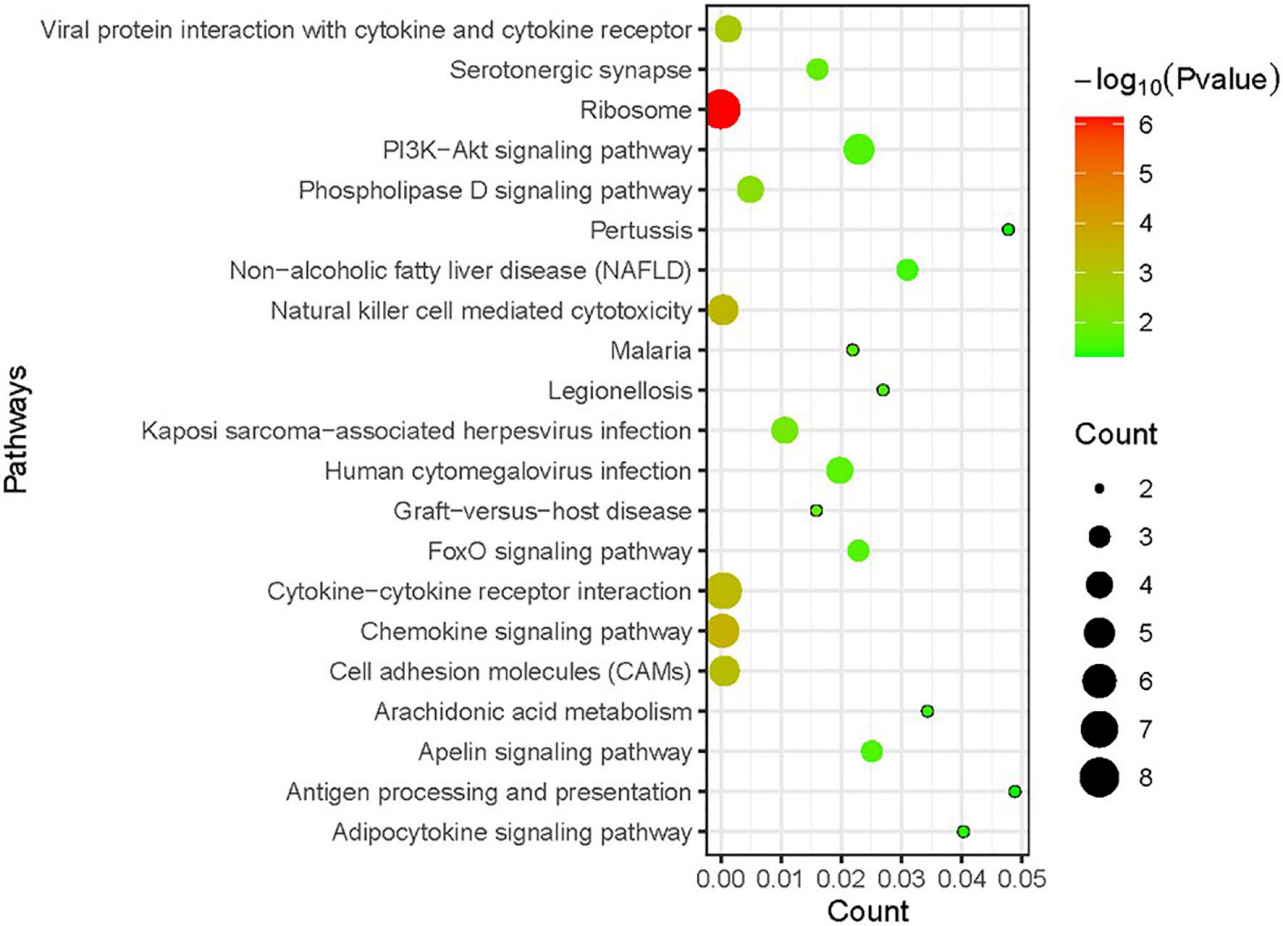
Kyoto Encyclopedia of Genes and Genomes (KEGG) enrichment analysis of DEGs in RSV-infected children compared with healthy children. The size of the circle indicates the gene number; the colour represents the log P values. P value <0.05 and FDR < 0.01 were used as the thresholds for pathway assignment. Note that most DEGs were enriched in immune-related pathways such as “chemokine signaling pathway”, “natural killer cell-mediated cytotoxicity” and “cytokine–cytokine receptor interaction”.

KEGG enrichment analysis was performed on DEGs to further investigate the related biological pathways (**Figure 3**). DEGs were significantly enriched in “ribosome” (P value=7.07E-07), followed by “chemokine signaling pathway” (P value=2.63E-04). Other enriched pathways included “natural killer cell mediated cytotoxicity”, “cytokine–cytokine receptor interaction”, and “cell adhesion molecules (CAMs)” (P value <1.00E-03).

### Differentially Expressed Genes Over Twofold Changes in Expression

**Figure 4:**
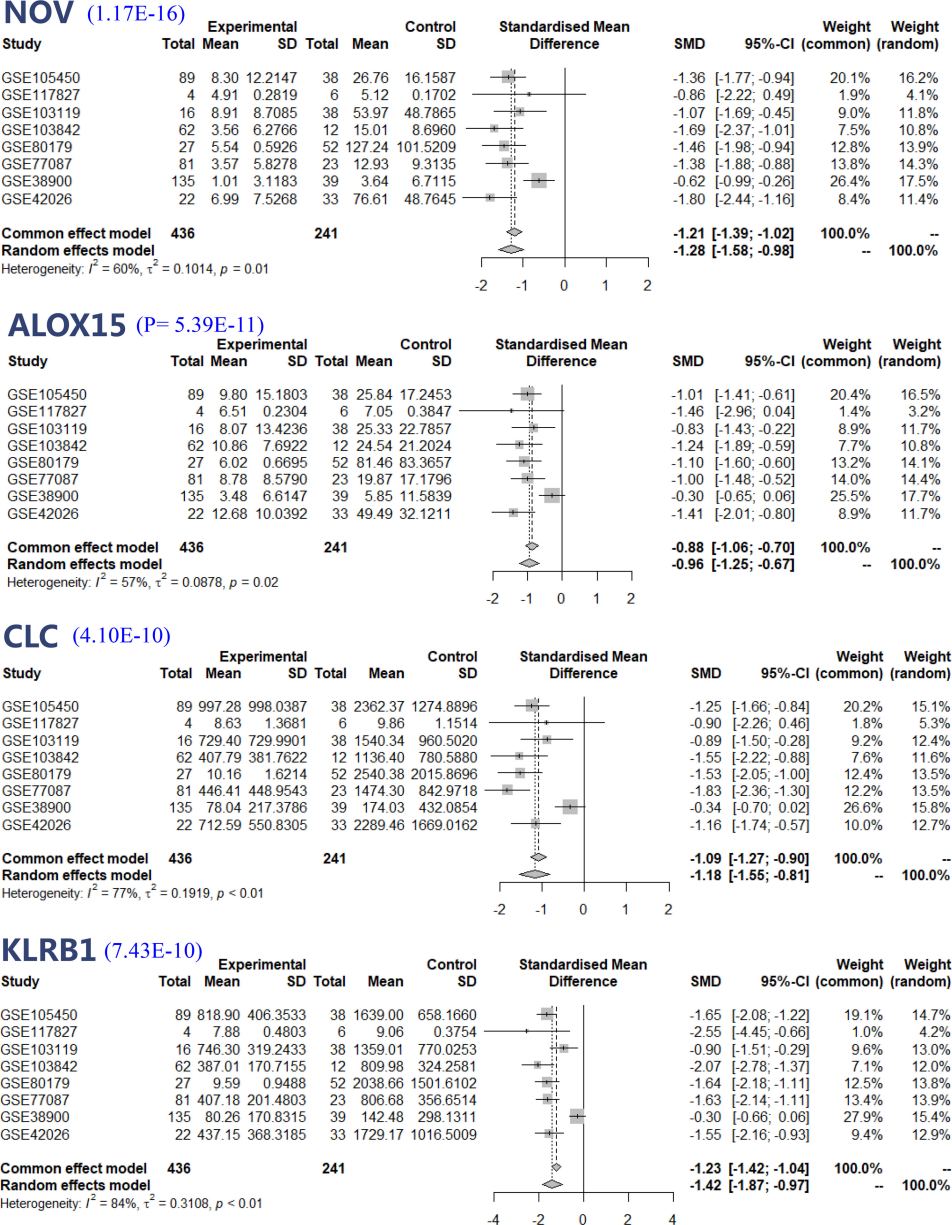
Forest plot based on random effect models of continuous variable meta-analysis.

DEG was screened for genes associated with RSV, and the results of a meta-analysis of the four genes with the most significant expression changes are reported in **Figure 4**, including *NOV*, a member of the CCN (Cyr61, CTGF, and NOV) family, with a P value of 1.17E-16, *Arachidonate 15-lipoxygenase* (*ALOX15*, P value=5.39E-11), *Charcot-Leyden Crystal Galectin* (*CLC*, P value=4.10E-10) and *Killer Cell lectin-like Receptor B1* (*KLRB1*, P value=7.43E-10). Based on the random effect model, the combined average difference and 95% confidence interval (CI) of the four most significant RSV-related genes were -1.28 (95% CI, -1.58 to - 0.98), -0.96 (95% CI, -1.25 to - 0.67), -1.18 (95% CI, -1.55 to -0.81), and -1.42 (95% CI, -1.87 to -0.97), respectively. Except for GSE117827, the mean fold changes of *NOV* and *CLC* in the experimental group were all downregulated by 2-fold or more than that in the control group. *ALOX15* was mostly downregulated by more than 2-fold in datasets GSE105450, GSE103119, GSE103842, GSE80179, GSE77087, and GSE42026, while *KLRB1* was mostly downregulated by more than 2-fold in datasets GSE105450, GSE103842, GSE80179, GSE77087, and GSE42026. Four of these genes had the largest changes in dataset GSE80179.

### Analysis of the Proportion of Different Immune Cell Types

**Figure 5:**
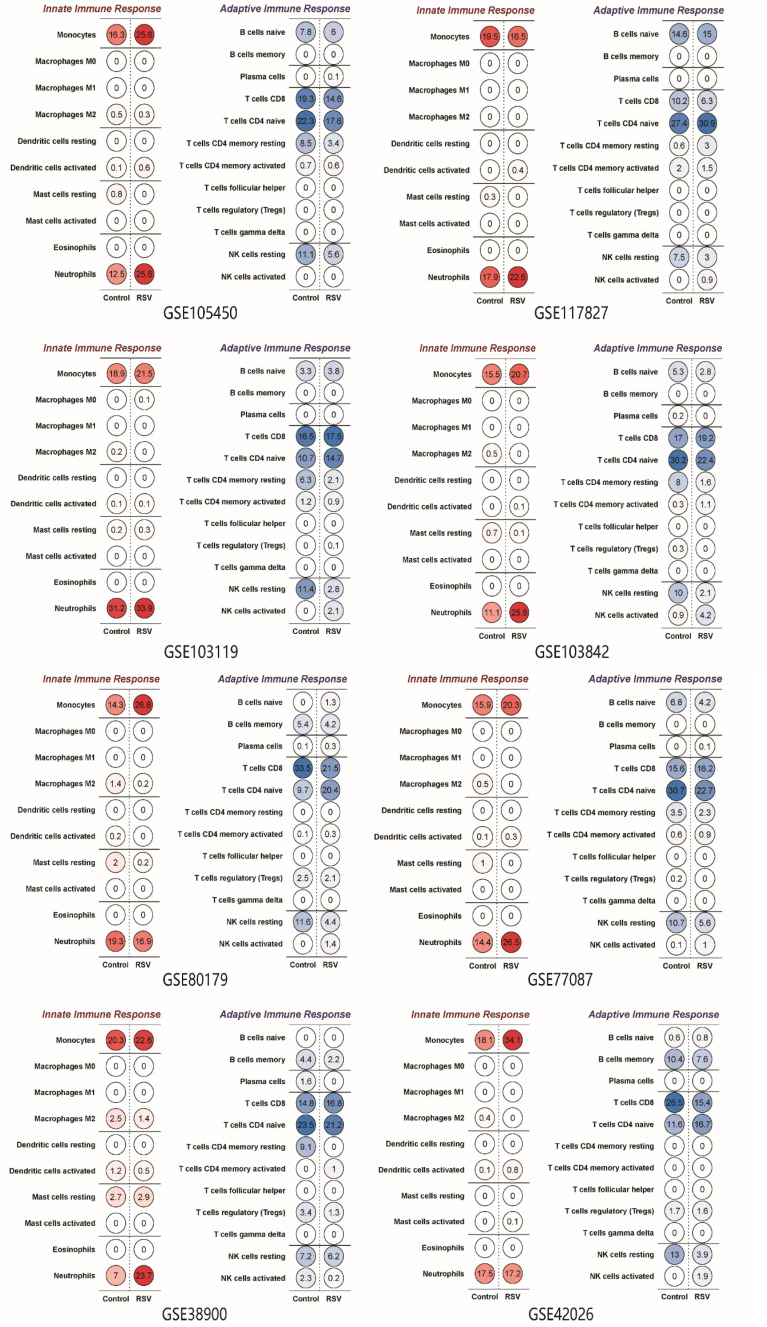
The proportion of immune cell types in different datasets. Note that the proportion of neutrophils in most datasets was higher in the RSV-infected groups than in the healthy groups.

The ratio of different immunocytes in whole blood was analysed using CIBERSORT, as shown in **Figure 5**. Monocytes, neutrophils, CD8 T cells and resting NK cells were the dominant cell types in the whole blood of the healthy groups and the RSV-infected groups. In 6 of the 8 datasets, the proportion of monocytes and neutrophils in children infected with RSV was higher than that in healthy controls. The proportion of resting NK cells in the RSV-infected groups was lower than that in the healthy control groups in the 7 datasets. The proportions of CD8 T cells and primordial B cells in the healthy groups and RSV-infected groups were variable.

## Discussion

RSV is the most important viral pathogen causing acute lower respiratory tract infection in children under 5 years old worldwide, and the primary viral factor leading to hospitalization for acute respiratory infection in infants under 1 year old seriously endangers children’s health (Shi et al., 2017). In this study, we conducted a meta-analysis of peripheral blood transcriptional microarray expression data from 436 children with RSV infection and 241 healthy children. A total of 319 DEGs were identified, of which the expressions of 46 genes were upregulated and the expressions of 273 genes were downregulated. Biological information databases, such as GO and KEGG, were applied to determine important signaling pathways related to immunity, such as the “chemokine signaling pathway”, “natural killer cell-mediated cytotoxicity” and “cytokine–cytokine receptor interaction”. In addition, CIBERSORT was used to determine the proportion of different immune cells in acute RSV infection and found that the proportion of neutrophils in the peripheral blood of most children with RSV infection was higher than that of healthy subjects.

In this study, *SIGLEC1* was the most upregulated gene and encodes a protein that is a lectin-like adhesion molecule that binds glycoconjugate ligands on the cell surface in a sialic acid-dependent manner (Crocker et al., 2007). Jans et al. identified that *SIGLEC1* was significantly upregulated in infants infected with RSV, and *SIGLEC1* was identified as the only gene that directly interacts with T cells (Jans et al., 2018). This study suggested that neonatal and adult primary T cells have low expression of the *SIGLEC1* receptor CD43, thus producing IFN-γ during RSV infection (Jans et al., 2018). However, the high expression of CD43 in adult memory T cells leads to the inhibition of IFN-γ release by *SIGLEC1*. In addition, monocytes have an inhibitory effect on T cells through *SIGLEC1* (Jans et al., 2018). This effect may also apply to other antigen-presenting cells, such as dendritic cells (DCs) and macrophages, which provides a basis for a better understanding of the antiviral immune response against RSV directly after birth.

*EMR4P* is associated with leukocyte adhesion (Kwakkenbos et al., 2004). In a study characterizing the expression of a novel whole-blood gene for asthma, dermatitis, and rhinitis in children and adolescents, *EMR4P* was consistently overexpressed in allergic diseases such as asthma, dermatitis, and rhinitis (Lemonnier et al., 2020). In addition, *EMR4P* has been identified as being involved in airway type 2 eosinophil inflammation (Ditz et al., 2021). In the results of this meta-analysis, *EMR4P* expression was lower in the RSV-infected group than in healthy children.

*TPT1P4* is a tumour protein. The TPT1/TCTP gene is a tumour protein that controls translation and encodes highly structured mRNA shielded by ribosomal protein, which is very similar to virus particles, activating protein kinase R (Amson et al., 2013). The results of our study showed that *TPT1P4* expression in the RSV-infected groups was lower than that in the healthy groups. The other two genes with the most significant expression changes were *RPS10P9* and *RPS7P11*, which were both related to ribosomes (Chen et al., 2007; Beabout et al., 2015) and had low expression in children with RSV infection.

According to our meta-analysis results, we identified four DEGs with fold changes and high significance, including *NOV*, *ALOX15*, *CLC* and *KLRB1*. All of these genes play functions related to viral infection. Among them, *NOV* is reported to be a biomarker of acute lung injury and is associated with inflammation and apoptosis of human alveolar epithelial cells (Zhu et al., 2020). *CLC* and *KLRB1* were identified to have downregulated expression in the whole blood of infants hospitalized with RSV (Fjaerli et al., 2006). Leonard’s study validated *CLC* and *ALOX15* as clinical biomarkers or risk factors for RSV infection in infants (Krilov et al., 2010).

The immunization of the pulmonary tissue with RSV and other respiratory viruses begins with the recruitment of immune cells from the blood into the lungs (Nuriev and Johansson, 2019). This inflammatory process is mainly controlled by chemokines. Many chemokines are produced during the infection process, and specific cell types are recruited through several unique chemokine receptors, such as CXCL1, CXCL2, and CXCL8, which can recruit neutrophils (Culley et al., 2006; Goritzka et al., 2014; Goritzka et al., 2015; Tang et al., 2016). CX3CL1 can recruit monocytes, NK cells and T cells (Johnson et al., 2012), and CXCL10 can recruit dendritic cells and T cells (Goritzka et al., 2014). During RSV infection, almost all chemokines are positively correlated with disease severity (Russell et al., 2017). An in-depth understanding of which cell types are the main sources of chemokines and how chemokine production is regulated will contribute to understanding the occurrence and maintenance of lung inflammation.

Cytokines are small secretory molecules that play a critical role in regulating the immune response and T-cell differentiation (Shachar and Karin, 2013). Several cell types can produce and secrete cytokines, including immune cells, epithelial cells, and endothelial cells (Kasahara, 2021). Cytokines are also considered to be key factors in establishing and regulating immune and inflammatory responses. In the natural immune stage of anti-RSV infection, RSV binds to Toll-like receptors on the surface of epithelial cells, which can promote the expression of proinflammatory cytokines such as IL-6 and TNF-α, leading to an inflammatory response (Kurt-Jones et al., 2000). RSV can promote Th2 cells to produce IL-4, IL-5, IL-9, IL-10, and IL-13, resulting in a strong Th2 cytokine response, inhibiting Th1-cell and Th1/Th2 immune imbalance (Openshaw and Chiu, 2013). Determining the specific cytokine expression profile after RSV infection will therefore provide effective immune strategies for anti-RSV treatment.

NK cells kill cells infected with pathogens through a range of mechanisms, mainly through the exocrine secretion of cytosolic particles and the involvement of extracellular death receptors (Smyth et al., 2005). NK-cell lytic particles containing perforin and granules enter infected cells after the formation of immune synapses and induce apoptosis through the caspase-3-mediated signaling pathway (Moretta et al., 2006; Lanier, 2008). The death receptor Fas ligand (FASL) on the infected cells interacts with the *tumour necrosis factor-related apoptosis-inducing ligand (TRAIL)* on NK cells, ultimately leading to target cell apoptosis (Lieberman, 2003). NK cells promote early innate immune responses by providing an early source of IFN-γ, activating T cells, and directly killing infected cells (Culley, 2009). However, there is evidence that after RSV infection, NK cells secrete a large number of cytokines, resulting in cytotoxicity and lung immune damage (Malloy et al., 2013; van Erp et al., 2019). In addition, the activating receptor *Natural Killer Group 2, Member D (NKG2D)*-mediated RSV infection increases the ability of NK cells to produce excessive IFN-γ, while IFN-γ can induce NK cells to activate T cells, which may contribute to lung injury during RSV infection (Liu et al., 2018).

Neutrophils are an essential part of the innate immune system, which plays a crucial role in identifying pathogens, killing invasive pathogens, presenting antigens to T cells, recruiting other inflammatory cells and producing cytokines (Mantovani et al., 2011; Metzler et al., 2011). RSV infection causes a strongly systemic and respiratory neutrophil response. Neutrophils are the major cell type in the bronchial lavage fluid of children with severe RSV bronchiolitis and mild infection (Everard et al., 1994). Compared with the uninfected control group, neutrophil elastase (NE) levels in nasal aspirate and serum of paediatric patients with acute RSV infection increased (Emboriadou et al., 2007). RSV virus particles stimulated human neutrophils *in vitro* and induced ROS-dependent extracellular DNA traps named NETs through *protein-arginine deiminase type 4 (PAD4)* citrullination and downstream activation of the PI3K/Akt, ERK and p38 MAPK pathways (Muraro et al., 2018). The deposition of the NET product in the culture medium can capture the RSV virus in NE- and MPO-coated DNA lattices, indicating that NETs have a beneficial antiviral effect (Muraro et al., 2018). However, in calves severely infected with bovine RSV, extensive airway obstruction is caused by luminal obstruction consisting of mucin, cell debris, and NETs (Cortjens et al., 2016). In addition, the results from other experimental systems, such as allergic asthma, suggest that NETs may aggravate immunopathology. Virus-associated exacerbations of asthma were attenuated when NETs were suppressed by blocking NE or degrading NETs with DNase (Toussaint et al., 2017). These results suggest that neutrophils play a complex role in RSV infection.

## Conclusion

This study combined the results of eight whole-blood microarray transcriptome studies associated with childhood RSV acute respiratory infection and identified 319 DEGs. Among the most significant genes, *SIGLEC1* interacts with T cells, and *EMR4P* is related to an allergic reaction. In addition, some immune-related pathways were identified, such as the “chemokine signaling pathway”, “natural killer cell-mediated cytotoxicity” and “cytokine–cytokine receptor interaction”. The ratio of neutrophils in most RSV infections was found to be higher than that in healthy children by immunocyte infiltration analysis. The identified immune characteristics are expected to deepen the understanding of the immune response to acute RSV infection in children.

## Data Availability Statement

The original contributions presented in the study are included in the article/**Supplementary Material**. Further inquiries can be directed to the corresponding authors.

## Author Contributions

QF performed the experiments and drafted the manuscript. SL, HL, and BY performed the data analysis. LX, XH, LH, and ZX participated in the study design and coordinated the drafting of the manuscript. All the authors read and approved the final manuscript.

## Funding

This work was funded by the National Natural Science Foundation of China (82172275) and the CAMS Innovation Fund for Medical Sciences (CIFMS, 2019-I2M-5-026).

## Conflict of Interest

The authors declare that the research was conducted in the absence of any commercial or financial relationships that could be construed as a potential conflict of interest.

The reviewer XL declared a shared affiliation with the authors LX, QF, ZX to the handling editor at the time of review.

## Publisher’s Note

All claims expressed in this article are solely those of the authors and do not necessarily represent those of their affiliated organizations, or those of the publisher, the editors and the reviewers. Any product that may be evaluated in this article, or claim that may be made by its manufacturer, is not guaranteed or endorsed by the publisher.
